# Evaluation of Treatment Strategies for Male Prolactin-Secreting Pituitary Neuroendocrine Tumors

**DOI:** 10.7759/cureus.54503

**Published:** 2024-02-20

**Authors:** Norihiko Saito, Nozomi Hirai, Yuki Koyahara, Sho Sato, Yu Hiramoto, Satoshi Fujita, Haruo Nakayama, Morito Hayashi, Satoshi Iwabuchi

**Affiliations:** 1 Neurosurgery, Toho University Ohashi Medical Center, Tokyo, JPN

**Keywords:** galactorrhea, cabergoline, male, prolactin-secreting, pitnets

## Abstract

Prolactin-secreting pituitary neuroendocrine tumors (PitNETs) are more common in women. Male patients may also have few symptoms and have macroadenomas extending outside the sella turcica. This study aimed to report the results of cabergoline treatment in male patients with prolactin-secreting PitNET. The study included nine male patients aged 26-65 years (median, 46 years) diagnosed with prolactin-secreting PitNETs. The age at onset, prolactin values, tumor size, symptoms, and treatment were assessed. The mean prolactin value at the initial presentation was 2734.6 ng/mL, and the mean maximum tumor diameter was 40.4 mm. Visual field disturbance was the most common symptom (44.4%), followed by headaches (33.3%), asymptomatic symptoms (11.1%), and galactorrhea (11.1%). Eight patients responded to cabergoline treatment with normalization of prolactin levels and tumor shrinkage. One patient did not respond to the cabergoline treatment and required surgical intervention. There were no cases of cerebrospinal fluid leakage. Cabergoline was found to be an effective treatment for male prolactin-secreting PitNETs.

## Introduction

Prolactin (PRL)-secreting pituitary neuroendocrine tumors (PitNETs) account for approximately 40% of all PitNETs and are the most common tumors of this type [1]. PRL-secreting PitNETs are more common in female patients and is often the cause of menstrual irregularities, galactorrhea, and infertility, while male patients lack symptoms such as dysmenorrhea or hypogonadism, making early-stage detection difficult [2-5].　

Dopamine agonists (DAs), such as cabergoline and bromocriptine, are first-line treatments for PRL-secreting PitNETs [6,7]. Cabergoline is the first-line treatment for PRL-secreting PitNETs, and in female patients who are often able to start treatment at an early stage, it is effective within a short period after initiation, with PRL normalization and tumor shrinkage rates of >90%. A clinical study demonstrating the efficacy of cabergoline therapy for PRL-secreting PitNETs was conducted in female patients. However, there are only a few case reports of male patients, and the optimal dosage and duration of therapy remain unclear [8-10]. Male patients are often found to have higher PRL levels at diagnosis than female patients or have macroadenomas that drain the optic nerve, hypothalamus, or pituitary gland or invade the cavernous sinus [8,10-12]. In daily clinical practice, there is still some confusion in deciding the indications for surgery, drug therapy, and other treatment strategies because these macroadenomas are often accompanied by complications, such as visual field and vision problems.

## Materials and methods

This retrospective study was conducted at the Toho University Ohashi Medical Center, Tokyo, Japan. It was approved by the Ethics Committee of the Toho University Ohashi Medical Center (approval number: H21047). Male patients diagnosed with PRL-secreting PitNETs who were under treatment at Toho University Ohashi Medical Center between 2002 and 2018 were included in the study.

Diagnosis was based on clinical and biochemical assessment, including a standard protocol for pituitary magnetic resonance imaging (MRI). All patients fulfilled the diagnostic criteria of a PRL-secreting PitNETs (i.e., elevated PRL levels without evidence of pituitary stalk compression, primary hypothyroidism or drug-induced hyperprolactinemia, and positive pituitary MRI scan). PRL level was measured using the electrochemiluminescence immunoassay method (normal value: 4.29-13.69 ng/mL). Patients without any available medical information were excluded from the study. A total of nine male patients fulfilled all criteria and were enrolled in the study.

Data collection

Data were collected from the medical records of the hospital and included patient age at diagnosis, gender, symptoms, serum PRL levels, tumor size, treatment modalities, drugs and doses administered, and duration of treatment.

Treatment protocol

Eight patients were treated with cabergoline therapy. Cabergoline was administered orally at a single starting dose of 0.25 mg for the first two weeks, followed by 0.5 mg once weekly. After two months of treatment, the dose was adjusted every two months based on serum PRL levels. In patients whose PRL levels did not normalize, cabergoline was increased to 3 mg weekly, and the dose was reduced in patients whose PRL levels decreased to less than 5 ng/L. Cabergoline was withdrawn only in patients whose PRL levels remained normal after the dose reduction.

Indication for surgery

In this study, surgical treatment was preferred when the patients had DA resistance (Case 8) and pituitary apoplexy (Case 9). DA resistance is defined as the failure to achieve normal PRL levels and failure to achieve tumor size reduction of at least 50% with maximal conventional doses of medication.

Statistical analysis

Data were analyzed using GraphPad Prism (Version 9; Dotmatics, Boston, Massachusetts, United States). Serum PRL values are presented as mean ± standard error (SE) unless otherwise noted.

## Results

This study included nine male patients with PRL-secreting PitNETs aged 26-65 years (median, 46 years) who were diagnosed and treated and a mean observation period of 59.9 ± 15.6 months. Baseline characteristics of the nine patients are summarized in Table 1. The most common symptoms leading to diagnosis were visual field disturbance (n=4, 44.4%), headache (n=3, 33.3%), and galactorrhea (n=1, 11.1%), and there was one asymptomatic case (11.1%). Treatment was started with cabergoline at a usual dose of 0.25 mg/week, except for one patient who had pituitary apoplexy prior to the start of treatment.

**Table 1:** Baseline characteristics in nine patients with a PRL-secreting PitNET PRL: prolactin; CAB: cabergoline; TSS: transsphenoidal surgery; PitNET: pituitary neuroendocrine tumor Reference range of PRL is 4.29–13.69 ng/mL

Case No.	Age (years)	Symptom	PRL(ng/ml)	Maximal tumor diameter(mm)	Treatment	Maximal dose of CAB (mg/week)	Response to CAB	PRL levels after one year	Duration of CAB therapy (mo)
Case 1	46	asymptomatic	2332.5	40.6	CAB	2	Responder	5.6	57
Case 2	58	headache	1485.2	41.5	CAB	2	Responder	28.3	73
Case 3	61	visual field disturbance	4833.9	53.5	CAB	2	Responder	7.6	156
Case 4	65	headache	1001.2	32.6	CAB	3	Responder	11.6	119
Case 5	36	visual field disturbance	7631.6	48	CAB	1	Responder	5.8	24
Case 6	47	visual field disturbance	1565.6	34	CAB	1	Responder	3.5	48
Case 7	26	galactorrhea	292.3	20	CAB	2	Responder	5.6	28
Case 8	27	visual field disturbance	4914.4	25.4	CAB+TSS	2	Non-Responder	5.5	7
Case 9	44	headache, pituitary apoplexy	4245.1	68	TSS+CAB	1	Responder	7.3	37

Tumor size and serum PRL level

All tumors were macroadenomas with a mean maximum tumor diameter of 40.4 ± 13.9 mm. One patient had a pituitary apoplexy. Optic chiasmatic consolidation was noted in eight patients (88.9%), and invasion into the cavernous sinus was noted in eight patients (88.9%), including bilateral invasion in three patients. Bone invasion and bone destruction were recorded in the clivus and sphenoid bones in four cases (44.4%). All patients had a mean PRL of 2734.6 ng/mL at the time of initial examination (Table 1).

Therapeutic effect of cabergoline

**Figure 1:**
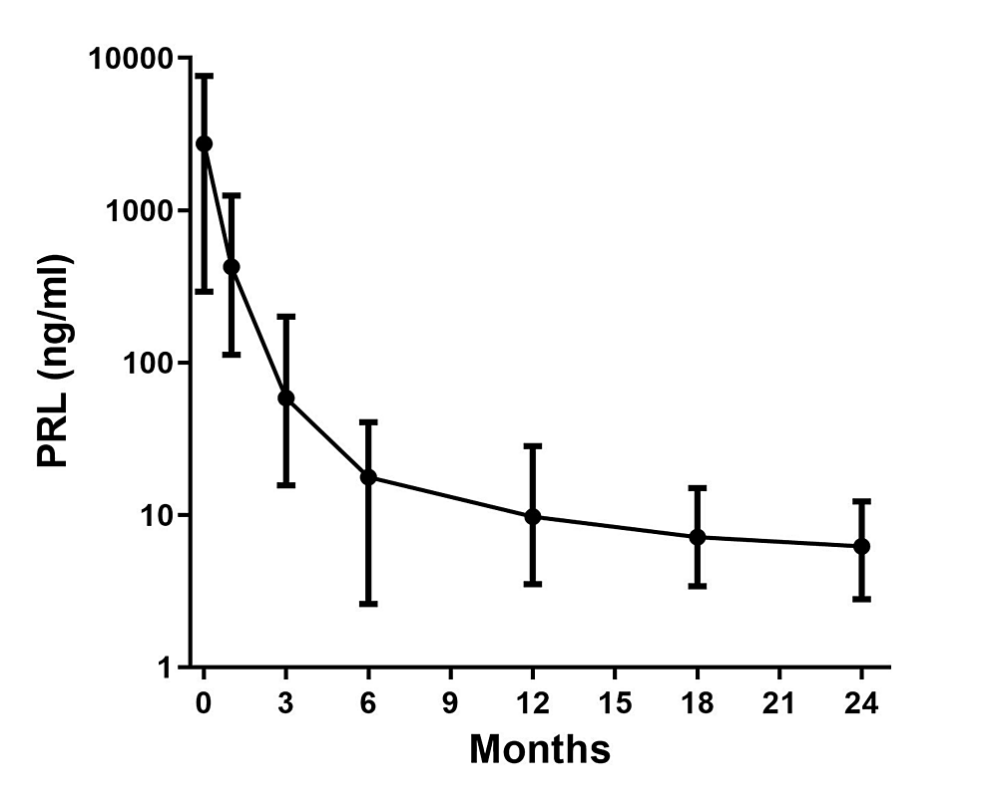
PRL suppression effect of cabergoline in male PRL-secreting PitNET (mean ± SE) Serum PRL of the seven patients treated with cabergoline monotherapy markedly decreased to 426.6 ± 369.6 ng/mL at one month, 58.4 ± 59.8 ng/mL at three months, and 9.71 ± 7.93 ng/mL at 12 months after starting oral cabergoline therapy. PRL: prolactin; PitNET: pituitary neuroendocrine tumor; SE: standard error Error bars indicate the standard error (SE) of the means.

The pretreatment PRL of the seven patients treated with cabergoline monotherapy was extremely high (3007 ng/mL), but it markedly decreased to 426.6 ± 369.6 ng/mL at one month, 58.4 ± 59.8 ng/mL at three months, and 9.71 ± 7.93 ng/mL at 12 months after starting oral cabergoline therapy (Figure 1). The overall PRL normalization rate after initiating cabergoline was 100%, the median time to PRL normalization was 24 weeks, and the mean maximum dose of cabergoline used during the course of the study was 1.56 ± 0.2 mg/week (Table 1). Side effects included headache and lightheadedness in only two patients; however, the symptoms resolved. Cerebrospinal fluid (CSF) leakage was not observed.

Two patients underwent surgical treatment: one with pituitary apoplexy and the other with poor response to cabergoline therapy. Patients with pituitary apoplexy were administered cabergoline therapy after surgery.

Representative cases

Case 7

**Figure 2:**
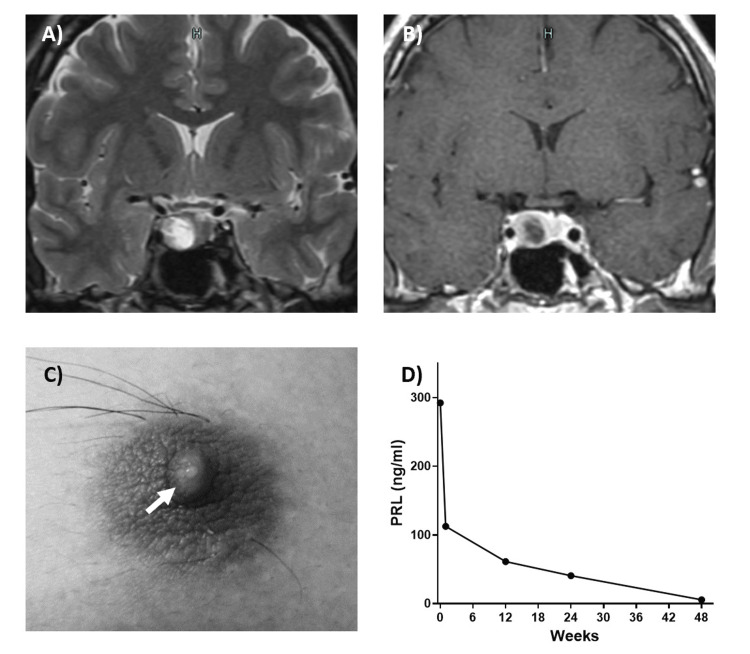
A case of a patient presenting with galactorrhea (Case 7) A: T2-weighted image, coronal section; B: T1 contrast image, coronal section. A neoplastic lesion is seen in the sella turcica; C: galactorrhea (arrow); D: Changes in PRL values. Cabergoline is causing a rapid improvement. PRL: prolactin

A 20-year-old man was referred to our hospital with suspected galactorrhea and gynecomastia. PRL was 292.3 ng/mL, and MRI of the head revealed a tumor-like lesion with a cystic portion 20 mm in the greatest diameter in the sella turcica. The patient was started on cabergoline (0.25 mg/week) with a diagnosis of PRL-producing PitNET. PRL quickly decreased, and galactorrhea disappeared. PRL levels remained normal with cabergoline at 2 mg/week (Figure 2).

Case 8

**Figure 3:**
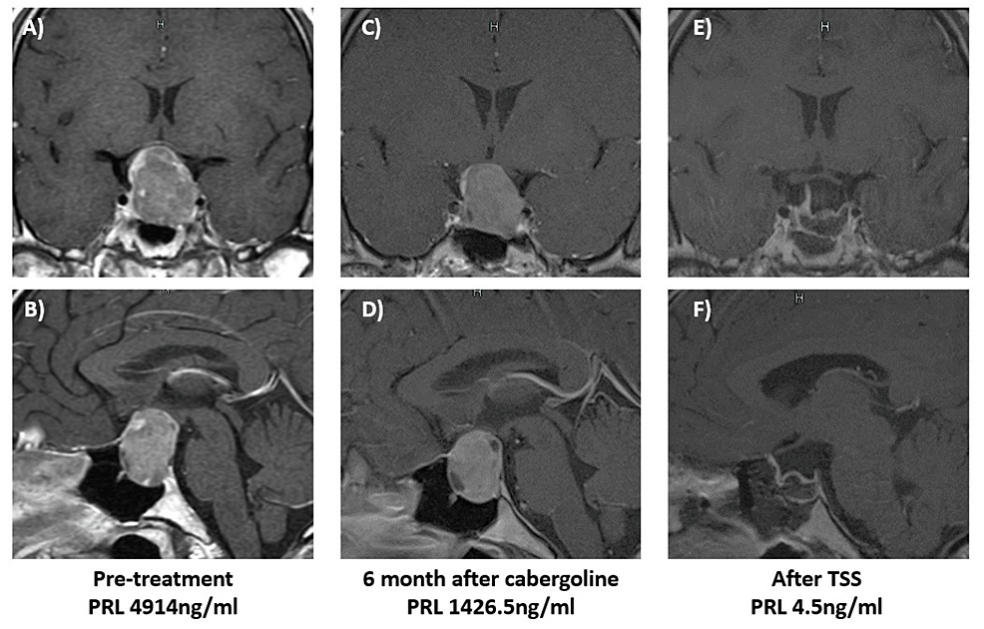
A patient who showed resistance to cabergoline therapy (Case 8) A, B: Pretreatment MRI scan. MRI scan of the head showed a tumor in the sella turcica, with a maximum diameter of 25 mm; C, D: After cabergoline treatment, PRL levels decreased, but there was no tumor shrinkage; E, F: Postsurgery. The tumor was completely removed, and the PRL has normalized. PRL: prolactin; TSS: transsphenoidal surgery

A 20-year-old man had been experiencing visual acuity loss and visual field disturbance for approximately one year. He visited a proximal ophthalmologist who detected bilateral hemianopsia and was referred to our hospital for further examination and treatment. The patient had a PRL of 4914 ng/mL, and an MRI scan of the head showed a tumor in the sella turcica, with a maximum diameter of 25 mm. Cabergoline (0.25 mg/week) was started to treat the PRL-producing PitNET, and the dose was increased to 2 mg/week; however, the PRL decreased to approximately 1400 ng/mL. The patient was refractory to cabergoline therapy and subsequently requested surgery, which was performed by transnasal endoscopic pituitary tumor resection. Postoperatively, the PRL level quickly decreased, and the patient did not require any drug therapy (Figure 3).

## Discussion

Male patients with prolactin-secreting PitNETs have symptoms such as decreased libido, and the tumors are often detected as large tumors at the time of diagnosis [3,4]. In our study, most tumors were >30 mm in diameter. When large tumors are accompanied by visual field impairment, treatment options, including surgical indications, are sometimes difficult to determine.

DAs are the first-line treatment for prolactin-secreting PitNETs, with cabergoline being the most potent, safe, and effective [6-8,13]. DAs not only decrease PRL secretion but also downregulate PRL gene expression in pituitary cells, resulting in a decrease in the number of PRL secretory granules, vacuolation of secretory cells [14], cell death including apoptosis [15], and bromocriptine-induced tumor fibrosis [16]. Thus, DAs have effects other than the suppression of PRL secretion and are thought to be a factor in the remission of prolactin-secreting PitNET after long-term use.

Colao et al. reported that cabergoline has an excellent outcome in microadenoma, with a PRL normalization rate of approximately 95% and a tumor shrinkage rate of more than 93 [8]. In our study, PRL was normalized in 89% of patients with a small dose of 0.5-3.0 mg/week. Cabergoline was also effective in shrinking tumors in 89% of the patients, suggesting that it is an excellent drug for treating large prolactinomas in male patients. Leong et al. reported that approximately 7% of patients with prolactin-secreting PitNETs treated with DAs developed CSF leakage [17]. In the present study, no CSF leakage was observed; however, since most male patients had macroadenomas, the possibility of CSF leak and other complications was high, and careful follow-up was needed.

Thus, cabergoline is very effective in male patients with prolactin-secreting PitNETs; however, Sarno et al. reported that approximately 18% of patients treated with cabergoline and 36% of those treated with bromocriptine had a poor response to prolactin-secreting PitNETs [18,19]. One of the nine patients in the current study underwent nasal endoscopic pituitary tumor resection because the tumor was refractory to treatment. The tumor was completely resected, and the PRL normalized without the need for adjuvant treatment. Prolactin-secreting PitNETs are invasive tumors that are difficult to treat with surgery alone [20]. In this case, cabergoline was administered preoperatively. Although sufficient tumor shrinkage was not achieved, the PRL value was reduced, suggesting that the added therapeutic effects, such as tissue changes due to partial cabergoline, enabled functional total resection. Therefore, surgical resection is considered for cabergoline-resistant cases [21,22].

Limitations of this study

Limited sample size may limit the generalizability of findings. The single-center approach may limit external validity. To increase the robustness and applicability of conclusions, future studies should consider employing larger, more diverse samples, incorporating control groups, and adopting a multicenter approach. These methods will help ensure that the findings are more generalizable and representative of the population.

## Conclusions

Cabergoline was highly effective in normalizing PRL and shrinking tumors in male patients with PL-secreting PitNETs, making it an excellent first-line treatment option. However, these patients often have macroadenomas, which can cause complications, such as CSF leakage during oral therapy; therefore, close follow-up is necessary. In addition, some cases were resistant to cabergoline treatment, and it was important to consider aggressive surgical treatment in these cases.
